# Risk factors for renal outcomes in children with antineutrophil cytoplasmic antibody-associated vasculitis: a nationwide retrospective study in China

**DOI:** 10.1007/s12519-023-00753-3

**Published:** 2023-10-19

**Authors:** Li-Wen Tan, Jun-Li Wan, Chun-Hua Zhu, Hong Xu, Zheng-Kun Xia, Li-Zhi Chen, Xiao-Chuan Wu, Fang Wang, Xiao-Rong Liu, Cheng-Guang Zhao, Xiao-Zhong Li, Jian-Hua Mao, Xiao-Wen Wang, Wen-Yan Huang, Yu-Hong Li, Jian-Jiang Zhang, Shi-Pin Feng, Jun Yang, Jiao-Jiao Liu, Chun-Lin Gao, Li-Ping Rong, Lan-Jun Shuai, Ke Xu, He-Jia Zhang, Qiu Li, Ai-Hua Zhang, Mo Wang

**Affiliations:** 1 Department of Nephrology, Children’s Hospital of Chongqing Medical University, Zhongshan 2nd Rd.136, Chongqing, 400014 China; 2 Ministry of Education Key Laboratory of Child Development and Disorders, National Clinical Research Center for Child Health and Disorders, China International Science and Technology Cooperation Base of Child Development and Critical Disorders, Children’s Hospital of Chongqing Medical University, Chongqing, China; 3 Chongqing Key Laboratory of Pediatrics, Children’s Hospital of Chongqing Medical University, Chongqing, China; 4 Department of Nephrology, Children’s Hospital of Nanjing Medical University, 72 Guangzhou Road, Nanjing, 210008 China; 5 Department of Nephrology, Children’s Hospital of Fudan University, National Paediatric Medical Center of China, Shanghai, China; 6 Department of Pediatrics, Jinling Hospital, Nanjing Medical University, Nanjing, China; 7 Department of Pediatrics, Jinling Hospital, The First School of Clinical Medicine, Southern Medical University, Nanjing, China; 8 Department of Pediatrics, Jinling Hospital, Medical School of Nanjing University, Nanjing, China; 9 Department of Pediatric Nephrology and Rheumatology, The First Affiliated Hospital of Sun Yat-Sen University, Guangzhou, China; 10 Department of Pediatrics, The Second Xiangya Hospital of Central South University, Changsha, China; 11 Department of Pediatrics, Peking University First Hospital, Beijing, China; 12 Department of Nephrology, Beijing Children’s Hospital, Capital Medical University, Beijing, 100045 China; 13 National Center for Children’s Health, Beijing, China; 14 Department of Pediatrics, Shengjing Hospital of China Medical University, Shenyang, China; 15 Department of Nephrology and Immunology, Children’s Hospital of Soochow University, Suzhou, China; 16 Department of Nephrology, Children Hospital, Zhejiang University School of Medicine, Hangzhou, China; 17 Department of Nephrology, Wuhan Children’s Hospital (Wuhan Maternal and Child Healthcare Hospital), Tongji Medical College, Huazhong University of Science & Technology, Wuhan, China; 18 Department of Nephrology and Rheumatology, Shanghai Children’s Hospital, School of medicine, Shanghai Jiao Tong University, Shanghai, China; 19 Pediatric Nephrology Department, Guiyang Maternal & Child Health Care Hospital, Guiyang, China; 20 Department of Pediatrics, The First Affiliated Hospital of Zhengzhou University, Zhengzhou, China; 21 Department of Nephrology, Chengdu Women and Children Central Hospital, Chengdu, 610041 China; 22 Department of Rheumatology and Immunology, Shenzhen Children’s Hospital, Shenzhen, China

**Keywords:** Antineutrophil cytoplasmic antibody, End-stage renal disease, Glomerulonephritis, Pediatric nephrology, Vasculitis

## Abstract

**Background:**

Pediatric antineutrophil cytoplasmic antibody-associated vasculitis (AAV) is a life-threatening systemic vasculitis featured by liability to renal involvement. However, there are few studies on the risk factors and predictive models for renal outcomes of AAV in children.

**Methods:**

Data from 179 AAV children in multiple centers between January 2012 and March 2020 were collected retrospectively. The risk factors and predictive model of end-stage renal disease (ESRD) in AAV were explored.

**Results:**

Renal involvement was the most typical manifestation (95.5%), and the crescent was the predominant pathological lesion (84.9%). The estimated glomerular filtration rate (eGFR) was evaluated in 114 patients, of whom 59.6% developed ESRD, and the median time to ESRD was 3.20 months. The eGFR [*P* = 0.006, odds ratio (OR) = 0.955, 95% confidence interval (CI) = 0.924–0.987] and the percentages of global glomerulosclerosis (pGGS; *P* = 0.018, OR = 1.060, 95% CI = 1.010–1.112) were independent risk factors for ESRD of renal biopsy. Based on the pGGS and eGFR at renal biopsy, we developed three risk grades of ESRD and one predictive model. The Kaplan‒Meier curve indicated that renal outcomes were significantly different in different risk grades (*P* < 0.001). Compared with serum creatinine at baseline, the predictive model had higher accuracy (0.86 versus 0.58, *P* < 0.001) and a lower coefficient of variation (0.07 versus 0.92) in external validation.

**Conclusions:**

Renal involvement is the most common manifestation of pediatric AAV in China, of which more than half deteriorates into ESRD. The predictive model based on eGFR at renal biopsy and the pGGS may be stable and accurate in speculating the risk of ESRD in AAV children.

Supplementary file 2 (MP4 18937 KB)

**Supplementary Information:**

The online version contains supplementary material available at 10.1007/s12519-023-00753-3.

## Introduction

Antineutrophil cytoplasmic antibody (ANCA)-associated vasculitis (AAV) is a rare autoimmune disease manifested by small vessel inflammation and cellulose necrosis [1]. As a life-threatening systemic disease, AAV can cause multiple organic injuries [2, 3], and ANCA-associated glomerulonephritis (AAGN) is a crucial factor determining the final prognosis of AAV patients, which may lead to the continuous deterioration of renal function. Eventually, approximately 20% of them develop end-stage renal disease (ESRD) [4].

Although childhood-onset AAV is rarer, kidney damage deteriorates more rapidly in children than in adults [2, 3]. It has been reported that 55%–58% of children with AAGN developed chronic kidney disease (CKD), and 29%–32% of patients progressed to ESRD [1, 5, 6]. Therefore, early diagnosis and precisely individualized treatment are crucial for improving renal function and reducing mortality in children. Berden’s classification categorizes the pathology of AAGN as focal, crescentic, sclerotic, or mixed lesions to predict renal outcomes from pathological lesions [7]. Previous research indicated that the clinical manifestations of AAV varied with diverse regions, races, and ages [1–3, 8], which makes it more difficult to implement these categories in pediatric patients. To date, few studies on pediatric AAV and AAGN have been conducted due to the rarity of AAV.

We retrospectively analyzed the clinical manifestations, renal pathologies, and renal outcomes in 179 Chinese patients with AAV from multiple centers. The factors resulting in adverse renal outcomes were further analyzed, and the predictive model for the prognosis of ESRD in pediatric AAGN was preliminarily explored.

## Methods

### Patients

This study was a multicenter retrospective cohort study. The enrolled centers are tertiary referral hospitals with a nephrology department or rheumatology department. A total of 179 children from 17 centers in China were recruited in the study from January 1st, 2012, to March 1st, 2020. Inclusion criteria were as follows: (1) children ≤ 18 years at initial diagnosis; (2) patients who met the 2007 European Medicines Agency classification [9] or 2012 Chapel Hill Consensus Conference definitions [10]. Patients with vasculitis caused by systemic lupus erythematosus, drugs, infections, or tumors were excluded.

The study was approved by the Ethics Committee of the Children’s Hospital of Chongqing Medical University (approval number: 149/2022) and other enrolled centers. This study was registered at the Chinese Clinical Trial Registry (registered number: ChiCTR2000034203).

### Data collection and definitions

The data from different centers were collected using a standard case report form from the electronic medical record system, and all collectors were finely trained. The clinical data included age, gender, manifestations, and laboratory investigations. Laboratory findings, including white blood cells, hemoglobin, platelets, C-reactive protein, albumin, immunoglobulin G, C3, and ANCA serological indicators were collected. ANCA-testing indicators included immunofluorescence for p-ANCA or c-ANCA, and enzyme-linked immunosorbent assay (ELISA) for myeloperoxidase-ANCA (MPO-ANCA) or proteinase 3-ANCA. Data related to renal involvement were analyzed, including serum creatinine (Scr), blood urea nitrogen (BUN), urine composition, and estimated glomerular filtration rate (eGFR). The eGFR was calculated using the Schwartz formula [11]. The pathological features of the kidney were collected retrospectively, including impairments of glomeruli, tubules, and interstitium. Normal glomeruli were defined as no scarring, crescents, or fibrinoid necrosis within the glomeruli. Sclerosis was divided into segmental glomerulosclerosis (< 50%) and global glomerulosclerosis (> 50%). The formula for percentage of normal glomeruli is the number of normal glomeruli/number of all glomeruli, and the formula for percentage of global sclerosis is the number of global sclerosis glomeruli/number of all glomeruli. The pediatric vasculitis activity score (PVAS) was used to assess initial vasculitis activity [12].

According to previous studies and PVAS, the definitions of AAGN are as follows [1, 12, 13]: (1) definite pathological lesion of kidney; (2) when kidney pathology was unavailable, AAGN was defined as hematuria (> 3 cells/high-power field, > 17 cells/μL on urinalysis, or red cell casts); proteinuria (≥ 0.15 g/24 hours, urine protein/Cr ≥ 2.0 mg/mg, or positive in urinalysis); renal insufficiency (rise in creatinine > 10% or eGFR fall > 25%) and rapidly progressive glomerulonephritis (RPGN). RPGN was defined as an eGFR decline of more than 50% in less than three months [1, 13]. The indications for renal biopsy included increased Scr, decreased eGFR, and persistent proteinuria or hematuria.

The eGFR of the patient was evaluated at the last follow-up to determine the stages of CKD according to the Kidney Disease: Improving Global Outcomes 2021 criteria [14]. Based on the eGFR at the endpoint, patients were divided into the ESRD (CKD stage 5) group and the non-ESRD (CKD stage 1–4) group. Further statistical analyses were performed to discover risk factors for ESRD.

**Fig. 1:**
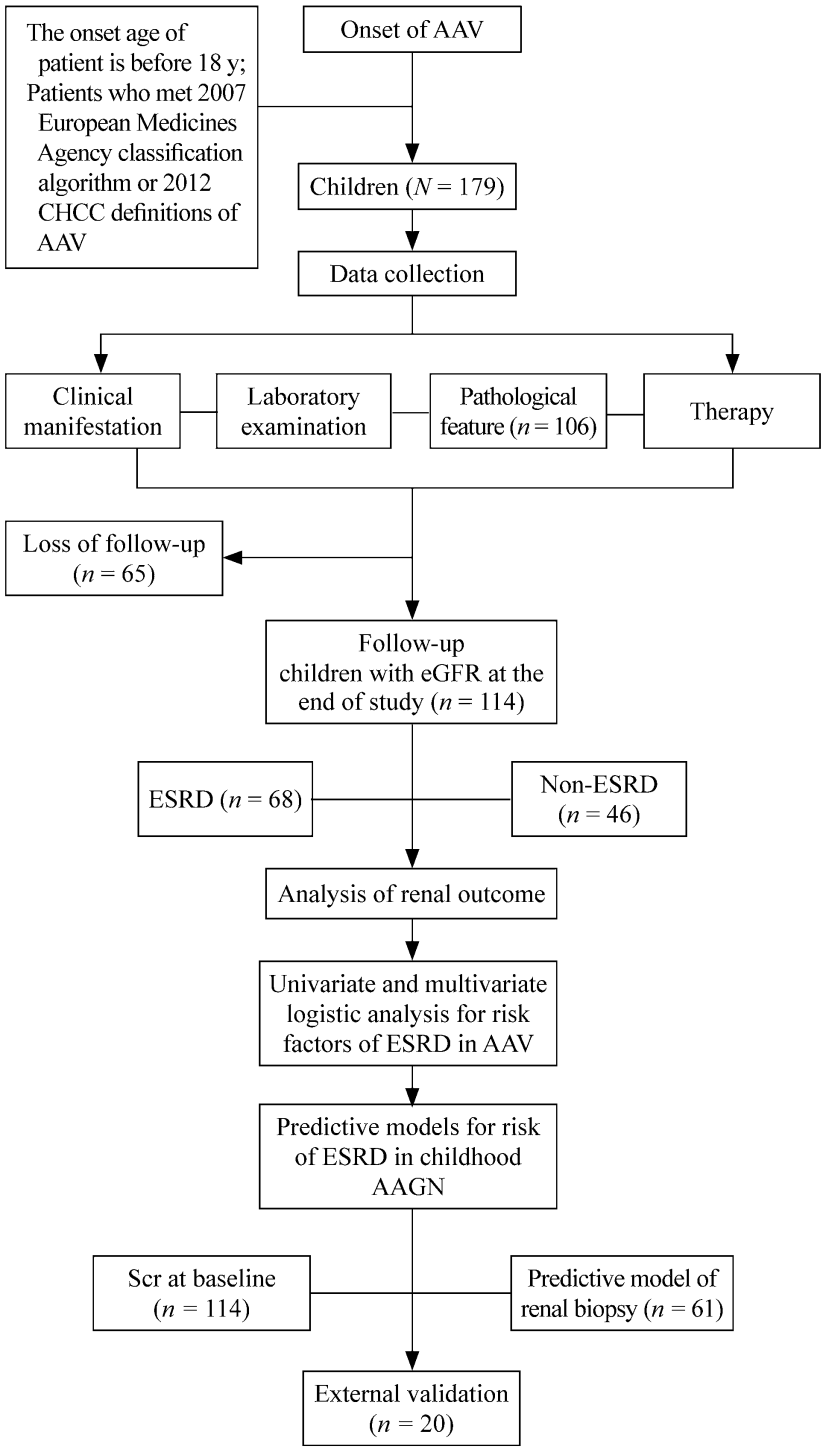
Flow chart of the study design. *CHCC* Chapel Hill Consensus Conference, *AAV* antineutrophil cytoplasmic antibody-associated vasculitis, *eGFR* estimated glomerular filtration rate, *ESRD* end-stage renal disease, *AAGN* antineutrophil cytoplasmic antibody-associated glomerulonephritis

The predictive model of ESRD in AAV was established with independent risk factors. Considering the impact of renal pathology on prognosis, the model was established according to both clinical data and pathological indexes. The study design is shown in Fig. 1.

### Statistical analysis

Continuous variables were expressed as the mean ± standard deviation (SD) or median with interquartile range and were analyzed by independent-sample *t* tests, analysis of variance, and Mann–Whitney *U* tests for comparison after checking the normality of the distribution with the Kolmogorov‒Smirnov test. Categorical variables were expressed as numbers or percentages and were analyzed with the Chi-squared test or Fisher’s exact test. Multivariate logistic regression analysis was performed to identify independent risk factors. Logistic regression results are shown with *P* values and odds ratios (ORs) with 95% confidence intervals (95% CIs), and a calculation formula for predicting probability was constructed with these independent risk factors. Kaplan‒Meier (KM) analysis was used for survival analysis. The area under the receiver operating characteristic (ROC) curve (AUC) was analyzed to assess the accuracy and to identify the cutoff point. A two-tailed *P* value < 0.05 was considered statistically significant. Multiple imputations were used to impute missing data. SPSS version 26.0 (IBM Corporation, Armonk, New York, USA) was used for statistical analysis. Figures were created with GraphPad Prism version 8.0 (GraphPad Software, San Diego, California, USA).

Model validation included internal and external validation. For internal validation, the validation patients obtained through sampling of 0.4–0.5 proportional samples from the overall patients at each time were substituted into the predictive model to obtain accuracy, and 30 times random sampling was carried out to analyze the stability of model internal validation. The repeated sampling method of bootstrap in internal validation was used to prevent overfitting of the predictive model by R 4.2.1, which can avoid bias due to the small sample size. For external validation, twenty children with AAV, from the Children’s Hospital of Chongqing Medical University and the Children's Hospital of Nanjing Medical University, were included. The accuracy of the predictive model was obtained after substituting the prognostic data, and the stability of the model external validation was verified by randomly selecting 0.4–0.5 proportional patients on 30 occasions. The predictive accuracy of random sampling was presented as the mean ± SD. The coefficient of variation (CV) was used to determine the discrete degrees of models and was calculated as SD/mean. The *t* test was used to compare the differences between the two models by MATLAB 2021a (Mathworks, New York, USA).

## Results

### Demographics, clinical manifestations, and laboratory characteristics at baseline

One hundred and seventy-nine children with AAV were included in this study, the number of cases from each center shown in Supplementary Table 1; 17.9% of patients were male, with a male-to-female ratio of 1:4.6. The median age at diagnosis was 10.1 (8.0–12.1) years. One hundred and thirty-six (76.0%) patients had microscopic polyangiitis (MPA; Supplementary Table 2), and most patients were MPO-ANCA (77.6%) and/or p-ANCA positive (60.3%, Table 1). Among 20 patients who had no ANCA results or were negative in ELISA, all of them had ANCA results in immunofluorescence (IF), and 16 patients had positive p-ANCA or c-ANCA. Four patients who had negative ANCA in ELISA and IF were diagnosed by renal biopsy, in which a renal pathology crescent was observed. A total of 114 patients had the last follow-up data, and the clinical manifestations of follow-up and missing follow-up patients were analyzed. Except for proteinuria and renal function, there was no significant difference in other clinical manifestations between the two groups, which also suggests that the missing follow-up patients had milder renal involvement (Supplementary Table 3).

**Table 1:** Laboratory parameters at baseline

Parameters	All (*N* = 179)	Follow-up patients (*n* = 114)	ESRD (*n* = 68)	Non-ESRD (*n* = 46)	*P*
WBC (× 10^9^/L), median (IQR)	8.34 (6.63–10.97)	8.38 (6.73–11.04)	8.38 (7.03–9.93)	8.44 (6.58–11.96)	0.701
Hemoglobin (g/L), median (IQR)	86.50 (68.75–107.25)	84.00 (66.00–101.00)	76.00 (60.50–90.50)	93.50 (78.25–116.00)	< 0.001
Albumin (g/L), median (IQR)	32.80 (28.90–37.70)	32.05 (27.62–37.00)	30.30 (25.00–34.00)	36.90 (31.60–40.20)	< 0.001
IgG (g/L), median (IQR)	9.83 (7.33–13.4)	9.35 (7.39–13.48)	9.00 (6.12–12.53)	10.59 (7.80–14.20)	0.121
C3 (g/L), median (IQR)	0.96 (0.79–1.11)	0.91 (0.74–1.10)	0.90 (0.73–1.06)	1.01 (0.76–1.14)	0.284
ANCA by ELISA, *n* (%)					
MPO-ANCA	139 (77.6)	91 (79.8)	58 (85.2)	33 (71.7)	Reference
PR3-ANCA	15 (8.4)	8 (7.0)	3 (4.4)	5 (10.9)	0.351
PR3-ANCA and MPO-ANCA	5 (2.8)	4 (3.5)	2 (2.9)	2 (4.3)	0.500
Negative	9 (5.1)	5 (4.4)	2 (2.9)	3 (6.5)	0.360
Missing	11 (6.1)	6 (5.3)	3 (4.4)	3 (6.5)	0.394
ANCA by IF, *n* (%)					
p-ANCA	108 (60.3)	71 (62.3)	46 (67.6)	25 (54.3)	Reference
c-ANCA	8 (4.5)	5 (4.4)	1 (1.5)	4 (8.7)	0.067
c-ANCA and p-ANCA	4 (2.2)	2 (1.8)	2 (2.9)	0 (0.0)	0.534
Negative	19 (10.6)	11 (9.6)	6 (8.8)	5 (10.9)	0.520
Missing	40 (22.3)	25 (21.9)	13 (19.1)	12 (26.1)	0.340
SBP, median (IQR)	120.00 (105.00–135.00)	123.00 (107.00–138.00)	132.00 (119.25–147.25)	110 (101.50–123.00)	< 0.001
24-h urinary protein (g/d), median (IQR)	1.31 (0.64–2.29)	1.49 (0.86–2.75)	1.80 (1.15–2.95)	1.10 (0.69–2.10)	0.055
BUN (mmol/L), median (IQR)	15.95 (5.47–30.03)	20.31 (8.58–31.66)	27.54 (17.65–37.23)	7.30 (4.21–16.15)	< 0.001
Scr (µmol/L), median (IQR)	195.00 (46.00–628.30)	426.40 (79.55–721.00)	651.00 (427.00–836.00)	76.80 (41.33–197.72)	< 0.001
eGFR (mL/min/1.73m^2^), median (IQR)	33.3 (11.2–135.6)	15.60 (10.45–77.20)	11.40 (8.20–15.30)	79.60 (27.03–159.03)	< 0.001
PVAS, median (IQR)	13.00 (12.00–18.00)	14.00 (12.00–18.00)	14.00 (12.00–18.00)	12.00 (12.00–17.25)	0.062

*ESRD* end-stage renal disease, *WBC* white blood cell, *IgG* immunoglobulin G, *ANCA* anti-neutrophil cytoplasmic antibodies, *ELISA* enzyme-linked immunosorbent assay, *MPO-ANCA* myeloperoxidase-ANCA, *PR3-ANCA* proteinase 3-ANCA, *IF* immunofluorescence, *SBP* systolic blood pressure, *BUN* blood urea nitrogen, *Scr* serum creatine, *eGFR* estimated glomerular filtration rate, *PVAS* paediatric vasculitis activity score, *IQR* interquartile range

Among 171 patients with renal involvement, the main manifestations were hematuria (28.5% gross hematuria), proteinuria (29.6% nephrotic syndrome proteinuria), edema (46.9%), and RPGN (32.4%) at baseline. Constitutional symptoms were the most common extrarenal manifestation (37.9%), followed by cutaneous involvement (22.9%) and respiratory system involvement (17.9%, Supplementary Table 4).

### Renal pathological characteristics and clinical features of renal biopsy

In this cohort, 106 patients with AAGN underwent renal biopsy, and there was no significant difference in clinical manifestations between children with and without renal pathology (Supplementary Table 5). As a retrospective study, 73 patients did not undergo pathological examination due to medical conditions or social factors. The median time from onset to diagnosis was 0.79 (0.33–2.24) months. Glomerular lesions were common, with a median normal glomerulus rate of 17.70% (0.00%–45.85%). The crescent was observed in 84.9% of patients, and the fibrocellular crescent was the predominant subtype. Renal tubulointerstitial damage was prevalent in AAGN patients, including interstitial inflammatory cell infiltration (87.7%), tubular atrophy (60.4%), and interstitial fibrosis (52.8%). Granulomas and necrosis (2.8%) were observed (Supplementary Table 6). The clinical features at biopsy, including proteinuria (94.3%), hematuria (92.8%), and decreased eGFR [36.93 (15.24–112.32) mL/minute/1.73 m^2^], are shown in Supplementary Table 6.

### Therapy and prognosis

**Fig. 2:**
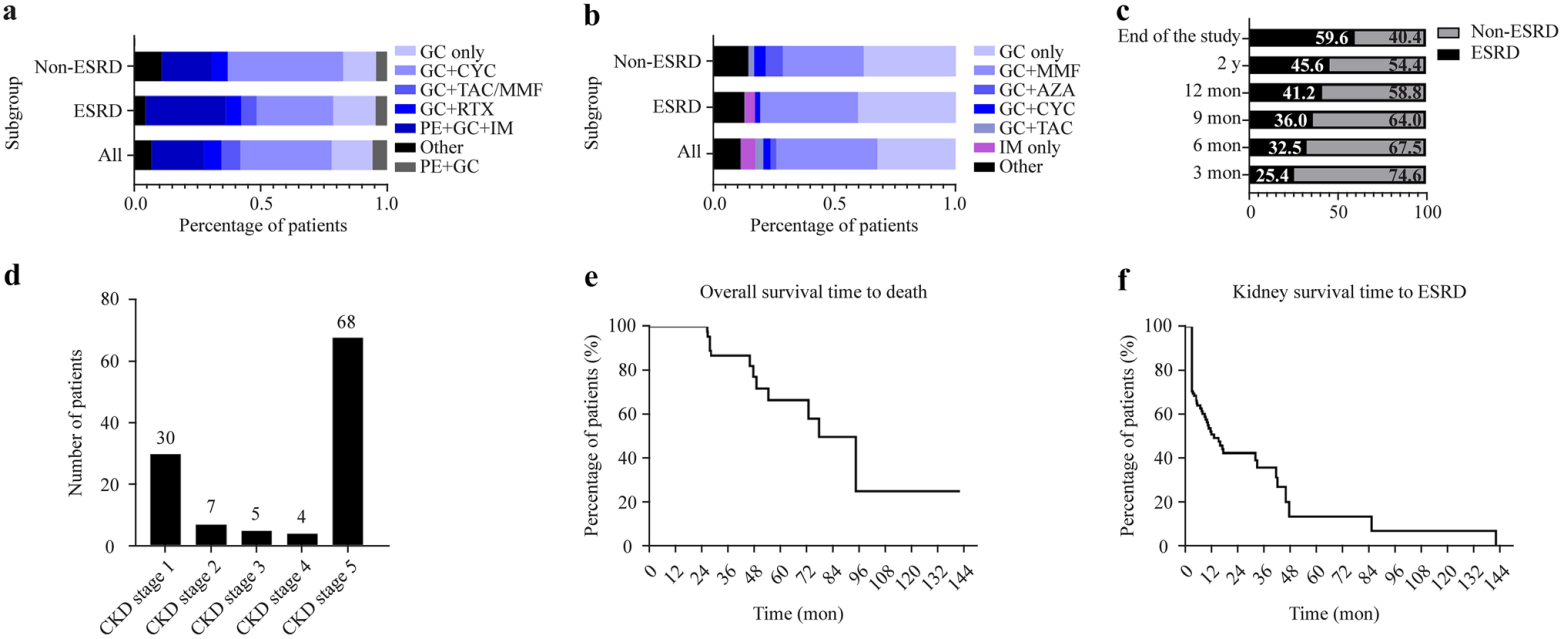
Therapeutic strategies and prognosis in AAV patients. **a** Immunosuppressive therapies for remission induction in 174 patients; **b** immunosuppressive therapies for remission maintenance in 127 patients; **c** percentage of ESRD and non-ESRD patients at different follow-up times; **d** numbers of patients in different CKD stages at the end of study; **e** Kaplan‒Meier curves of the time from diagnosis to death in 179 patients of the whole study; **f** Kaplan‒Meier curves of the time from diagnosis to ESRD in 114 patients. *AAV* antineutrophil cytoplasmic antibody-associated vasculitis, *GC* glucocorticoids, *CYC* cyclophosphamide, *TAC* tacrolimus, *MMF* mycophenolate mofetil, *RTX* rituximab, *PE* plasma exchange, *IM* immunosuppressor, *AZA* azathioprine, *ESRD* end-stage renal disease, *CKD* chronic kidney disease

Except for four patients who gave up treatment and one who received dialysis only, the therapeutic regimens of 174 patients in remission-induction were collected. Glucocorticoids (GCs; 97.7%) were widely used, where cyclophosphamide (36.2%) was the most common immunosuppressant, whereas 12 (6.9%) patients were combined with rituximab (Fig. 2a). Forty-seven patients were lost during follow-up until March 2020. In 127 patients with remission-maintenance, GC combined with mycophenolate mofetil (41.7%) was the most common therapy strategy, as only three patients received GC combined with azathioprine (Fig. 2b).

Fifteen (8.4%) patients died at the endpoint, with a median time to death of 46.03 (27.75–63.74) months. The causes of death included cerebral hernia, infection, cardiac failure, pulmonary hemorrhage, and respiratory failure (Fig. 2e). At the endpoint (March 2020), 114 of 179 AAGN patients were followed up with renal function, of which 65 were lost. The median follow-up time was 11.7 (3.52–27.83) months. Among the 114 patients, ESRD increased gradually (Fig. 2c). At the end of follow-up, 77 (67.5%) progressed to CKD stage 3–5, and 68 (59.6%) patients reached ESRD (Fig. 2d). Patients showed rapid loss of kidney function in the first year, with a median time to ESRD of 3.20 (3.00–12.33) months (Fig. 2f).

### Risk factors for renal outcomes

To identify the risk factors for ESRD, the variables of 114 patients at baseline were analyzed. The logistic regression analysis showed that the age at diagnosis, edema, RPGN, hemoglobin, platelet, albumin, systolic blood pressure, diastolic blood pressure, Bun, Scr, and eGFR at baseline were significantly different between the ESRD group (*n* = 68) and non-ESRD group (*n* = 46). Scr at baseline (*P* < 0.001, OR = 1.006, 95% CI = 1.004–1.009) was an independent risk factor for ESRD through multivariate logistic analysis (Table 2). There was no significant difference in plasma exchange between the ESRD group and the non-ESRD group (*P* = 0.214, Fig. 2a). There were 32 patients with eGFR > 60 mL/minute/1.73 m^2^ at baseline, and five patients achieved ESRD at the end of follow-up. There was no significant difference between the ESRD group and the non-ESRD group (*n* = 27, Supplementary Tables 7–9).

**Table 2:** Logistic regression analysis of risk factors of ESRD in childhood AAV

Variables	*P* value in univariate logistic analysis	OR (95% CI)	*P* value in multivariate logistic analysis	OR (95% CI)
Patients at baseline (*n* = 114)				
Age at diagnosis	0.043	1.127 (1.004–1.265)		
Edema	< 0.001	4.472 (2.001–9.995)		
RPGN	0.023	2.677 (1.145–6.261)		
Hemoglobin	< 0.001	0.968 (0.951–0.986)		
Platelet	< 0.001	0.993 (0.989–0.996)		
Albumin	< 0.001	0.828 (0.762–0.900)		
SBP	< 0.001	1.069 (1.040–1.100)		
DBP	< 0.001	1.082 (1.046–1.119)		
BUN	< 0.001	1.103 (1.058–1.148)		
Scr	< 0.001	1.006 (1.004–1.008)	< 0.001	1.006 (1.004–1.009)
eGFR at baseline	< 0.001	0.963 (0.945–0.981)		
Patients at renal biopsy (*n* = 61)				
Edema	0.035	3.382 (1.087–10.528)		
Oliguria/anuria	0.008	6.481 (1.620–25.927)		
Hemodialysis	0.005	4.773 (1.593–14.302)		
Platelet	0.006	0.992 (0.986–0.998)		
BUN	0.017	1.064 (1.011–1.120)		
Scr	< 0.001	1.005 (1.002–1.008)		
eGFR	0.004	0.960 (0.934–0.987)	0.006	0.955 (0.924–0.987)
24-h urinary protein	0.082	1.781 (0.930–3.410)		
Percentage of normal glomerulus	0.001	0.948 (0.918–0.979)		
Percentage of global glomerulosclerosis	0.003	1.038 (1.013–1.063)	0.018	1.060 (1.010–1.112)

*ESRD* end-stage renal disease, *AAV* antineutrophil cytoplasmic antibody associated vasculitis, *OR* odds ratio, *CI* confidence interval, *RPGN* rapidly progressive glomerulonephritis, *SBP* systolic blood pressure, *DBP* diastolic blood pressure, *BUN* blood urea nitrogen, *Scr* serum creatine, *eGFR* estimated glomerular filtration rate

Among 106 patients who underwent renal biopsy, 61 patients estimated the renal prognosis with eGFR at the end of follow-up, of which 52.4% (32/61) reached ESRD. Logistic regression analysis showed that edema, oliguria/anuria, hemodialysis, platelet count, BUN, Scr, eGFR, 24-hour urine protein, percentage of normal glomeruli, and percentage of global glomerulosclerosis (pGGS) on renal biopsy were associated with ESRD (Table 3). The eGFR at renal biopsy (*P* = 0.006, OR = 0.955, 95% CI = 0.924–0.987) and the pGGS (*P* = 0.018, OR = 1.060, 95% CI = 1.010–1.112) were independent risk factors for ESRD via multivariate logistic analysis (Table 2).

**Table 3:** Significant different clinical manifestations, laboratory parameters and pathological features of 61 patients at renal biopsy

Variables	Follow-up patients (*n* = 61)	ESRD (*n* = 32)	Non-ESRD (*n* = 29)	*P*
Edema, *n* (%)	21 (34.4)	15 (46.9)	6 (20.7)	0.032
Oliguria/anuria, *n* (%)	17 (28.3)	14 (43.8)	3 (10.7)	0.005
Hemodialysis, *n* (%)	29 (48.3)	21 (65.6)	8 (28.6)	0.004
Platelet (× 10^9^/L), median (IQR)	280.00 (195.00–348.00)	231.00 (170.00–288.00)	301.00 (234.00–403.00)	0.003
BUN (mmol/L), median (IQR)	16.60 (8.36–27.72)	26.40 (14.42–32.55)	8.82 (6.80–16.90)	0.001
Scr (µmol/L), median (IQR)	337.00 (97.10.00–543.66)	464.00 (347.30–683.50)	98.55 (57.60–226.00)	< 0.001
eGFR (mL/min/1.73 m^2^), median (IQR)	19.42 (13.10–72.36)	15.24 (11.04–18.16)	72.36 (29.93–121.67)	< 0.001
24-h urinary protein (g/d), median (IQR)	1.70 (1.10–2.22)	2.12 (1.46–3.38)	1.31 (0.84–1.71)	0.024
Normal glomerulus (%), median (IQR)	14.30 (0.00–38.80)	0.00 (0.00–11.63)	30.00 (16.70–56.50)	< 0.001
Global glomerulosclerosis (%), median (IQR)	11.60 (0.00–44.40)	33.15 (5.58–61.43)	2.00 (0.00–17.50)	0.002

*ESRD* end-stage renal disease, *BUN* blood urea nitrogen, *Scr* serum creatine, *eGFR* estimated glomerular filtration rate, *IQR* interquartile range

### Establishment and validation of the predictive model for ESRD in AAV

**Fig. 3:**
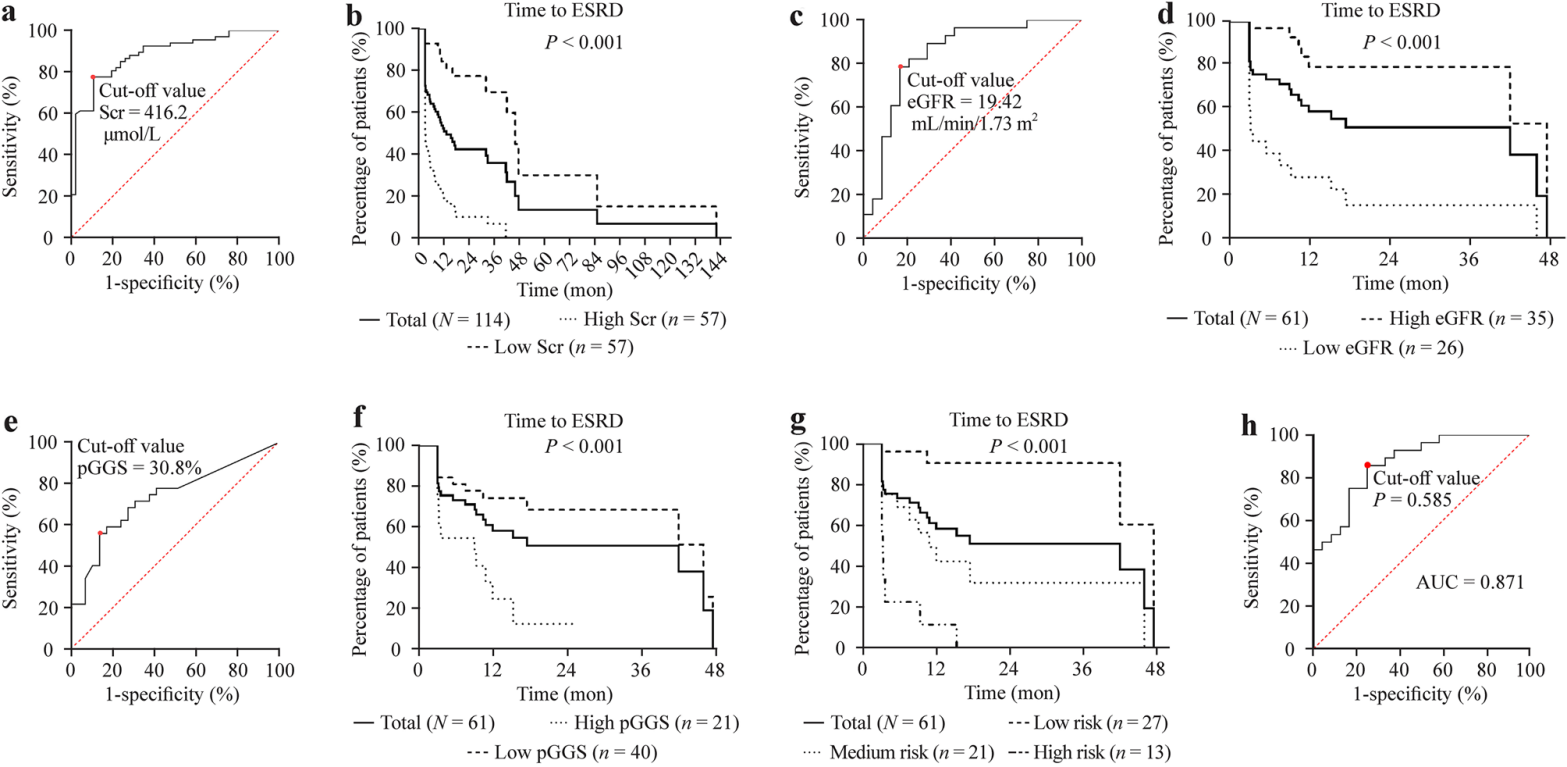
Receiver operating characteristic (ROC) curves and Kaplan‒Meier (KM) curves of survival analysis of risk factors in AAV children.** a** ROC of Scr at baseline for predicting ESRD in 114 AAV patients; **b** KM curve of the time to ESRD in patients with different Scr levels at baseline; **c** ROC of eGFR at renal biopsy for predicting ESRD 61 AAV in patients; **d** KM curve of the time to ESRD in patients with different eGFR levels at renal biopsy; **e** ROC of percentage of global glomerulosclerosis at renal biopsy for predicting ESRD 61 AAV in patients; **f** KM curve of the time to ESRD in patients with different pGGS at renal biopsy; **g** KM curve of the time to ESRD in patients with different risk groups; **h** ROC of *P* value of ESRD in the predictive model. *Scr* serum creatinine, *ESRD* end-stage renal disease, *eGFR* estimated glomerular filtration rate, *AAV* antineutrophil cytoplasmic antibody associated vasculitis, *AUC* area under the receiver operating characteristic curve, *pGGS* percentages of global glomerulosclerosis

As an independent risk factor for ESRD, the optimal cutoff value of Scr at baseline was 416.2 μmol/L, with an AUC of 0.885 in the ROC curve (Fig. 3a). Via the cutoff value, 114 children were divided into the high-Scr group and the low-Scr group, with a statistically significant difference in prognosis (*P* < 0.001, Fig. 3b). In addition, the cutoff value of eGFR at renal biopsy was 19.42 mL/minute/1.73 m^2^ with an AUC of 0.842, and the cutoff value for the pGGS was 30.8% with an AUC of 0.734 (Fig. 3c, e). The prognosis was different between different eGFR groups and different pGGS groups (*P* < 0.001, Fig. 3d, f). According to the eGFR and pGGS, patients were divided into the low-risk group (no risk factor), the medium-risk group (1 risk factor), and the high-risk group (2 risk factors), with significant differences in the renal outcomes (*P* < 0.001, Fig. 3g).

**Fig. 4:**
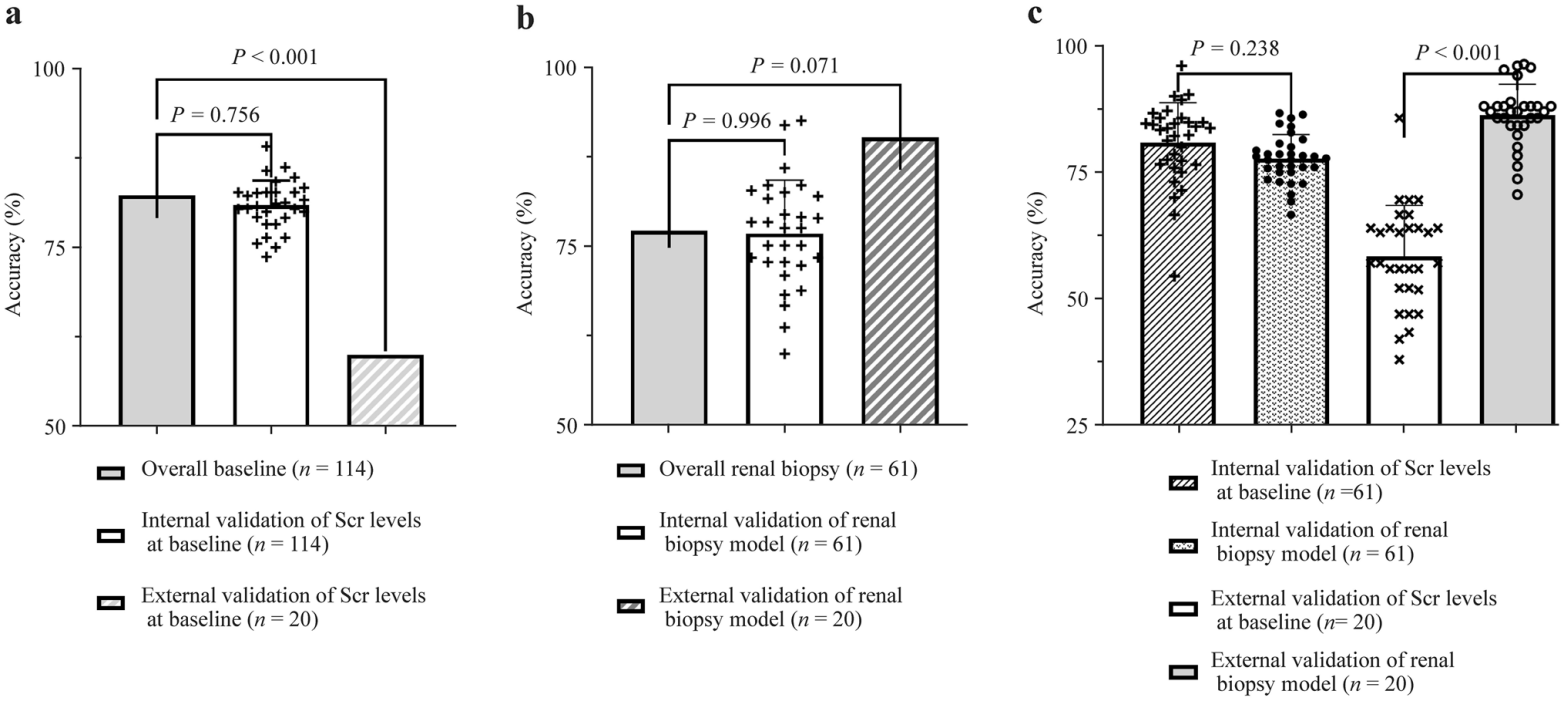
Comparison results of serum creatinine (Scr) at baseline and the predictive model. **a** The accuracy and stability of Scr at baseline in internal validation and external validation; **b** the accuracy and stability of the predictive model in internal validation and external validation; **c** the accuracy and stability between Scr at baseline and the predictive model in internal validation and external validation

Thus, based on eGFR at renal biopsy and the pGGS, we developed a model to predict the probability of ESRD. In this model, the probability of ESRD of renal biopsy is P = 1/[1 + exp(0.997–0.046 × eGFR + 0.058 × pGGS)], and the cutoff value for the model was 0.585 with an AUC of 0.871 (Fig. 3h). Furthermore, we compared the accuracy and stability of Scr at baseline and the model of renal biopsy in predicting ESRD. The Scr at baseline of 114 patients was used for internal validation to assess the accuracy of Scr in predicting prognosis, and 45–60 patients were randomly selected. Among 61 patients with renal pathology, 10–35 patients were randomly selected for the predictive model internal verification, and the stability of model internal verification was obtained through 30 random sampling. Twenty patients with complete follow-up and pathological data, from the Children’s Hospital of Chongqing Medical University and the Children’s Hospital of Nanjing Medical University, were enrolled in external validation, and 8–13 patients were randomly selected 30 times for verification to analyze the stability of model external verification. The clinical characteristics were similar in the internal and external validation cohorts (Supplementary Tables 10–11). Through 1000 repeated samples, the results of bootstrap showed no severe overfitting of either Scr (AUC = 0.89, sensitivity = 0.80, specificity = 0.80) or the predictive model (AUC = 0.88, sensitivity = 0.71, specificity = 0.84). Furthermore, the accuracy of Scr was higher in internal validation than in external validation (*P* < 0.001, Fig. 4a), while there were no differences in the model (*P* = 0.071, Fig. 4b). Regarding stability, both Scr and the model were stable in internal validation by random sampling (Scr *P* = 0.756 versus model *P* = 0.996). In internal and external validation by random sampling, there was no difference in the accuracy of Scr (*P* = 0.238, Fig. 4c), while the accuracy of the model in external validation was higher than that in internal validation (*P* < 0.001, Fig. 4c). In external validation, the CV of model 2 (CV = 0.07) was lower than that of Scr (CV = 0.92).

## Discussion

AAV is a systematic severe autoimmune vasculitis with multiorgan involvement, categorized as granulomatosis with polyangiitis (GPA), MPA, and eosinophilic GPA [10]. To date, there have been a few large studies of pediatric AAV, and even fewer studies have focused on AAGN [1, 8, 15, 16].

One hundred and seventy-nine AAV patients from 17 centers in China were enrolled in our study. Similar to previous studies, our study showed that pediatric AAV usually occurred in adolescence, which was more common in females and more prevalent in MPA [8, 16–18]. Compared with 45%-80% in other studies [1, 8], only 17.9% of the children in our study had respiratory tract involvement, probably due to respiratory involvement limited AAV patients might be misdiagnosed, or the lower incidence of respiratory tract involvement in MPA.

AAGN is a substantial characteristic of multisystem damage in AAV [15, 16], and the proportion of renal involvement was also significantly higher in previous MPA-predominated research [8, 15] than in GPA-predominated research [16]. We also found that up to 95.5% of children with AAV had AAGN, presenting various types of renal damage. It has been reported that pediatric AAGN has severe initial renal damage and rapid deterioration of renal function [1, 5, 6]. In this cohort, 32.4% of patients were diagnosed with RPGN at baseline, and more than 40% were carried out on hemodialysis as initial therapy, which is similar to previous reports. We considered that the inconsistencies between the number of cases of edema and the number of cases of proteinuria with nephrotic syndrome in children with AAV may be attributed to edema due not only to protein loss but also to decreased eGFR. Unexpectedly, the incidence of ESRD in pediatric AAGN is significantly higher both in our study and in several single-center studies in China than in other studies, which is up to 59.6% [18, 19]. On the one hand, based on a French retrospective multicenter study in pediatric AAV that indicated that non-Caucasian ethnicity was correlated with a higher risk of ESRD compared with Caucasian ethnicity [15], we presume that the high incidence of ESRD in Chinese children may also be caused by ethnic specificity. On the other hand, we speculate that the rapid progression of ESRD in Chinese children was due to the universality of crescent lesions.

To find applicable models to predict renal outcomes, we explored the risk factors for ESRD at the time of onset and renal biopsy. Ultimately, Scr at baseline, eGFR at renal biopsy, and pGGS were confirmed as independent risk factors for ESRD via multivariate logistic analysis. Through the cutoff value of the independent two risk factors for renal biopsy, patients were divided into different risk groups (Table 4). The KM curves in AAV showed different renal outcomes in different risk groups (Fig. 3), indicating the importance of the independent risk factors. As direct indicators of renal function, previous studies have shown that eGFR and Scr could affect renal prognosis [1, 15, 20]. Although Burden classification of AAGN cannot accurately predict renal prognosis [1, 21], renal pathology still plays an essential role in renal outcomes, including the percentages of normal glomeruli, the extent of interstitial fibrosis, and tubular atrophy [20, 22]. Our study showed that the pGGS was an independent risk factor for ESRD. GGS is characteristic of chronic kidney disease [23]. Excessive GGS means irreversible changes in the kidney.

**Table 4:** Pediatric renal risk grade of antineutrophil cytoplasmic antibody associated vasculitis

eGFR at renal biopsy ≤ 19.42 mL/min/1.73 m^2^	Percentages of global glomerulosclerosis ≥ 30.8%	Risk groups
−	−	Low risk
−	+	Medium risk
+	−	
+	+	High risk

*eGFR* estimated glomerular filtration rate. “+” positive, “−” negative

Due to the poor renal outcome of AAGN in children, there are few reliable models to predict ESRD. Brix et al. found that eGFR at diagnosis, percentage of normal glomeruli, tubular atrophy, and interstitial fibrosis could predict the renal outcome of AAV in adults [24]. We established a model to predict the risk of ESRD in pediatric AAV based on eGFR at renal biopsy and the pGGS with high accuracy (AUC = 0.871). AAV patients with a probability of more than 0.585 in the model were ascertained as high risk for ESRD. Since some patients were lost to follow-up or did not undergo renal biopsy, random sampling in the external validation and bootstrap in internal validation were used to prevent overfitting of the predictive model, which can avoid bias due to the small sample size. Compared with Scr at baseline, the predictive model had higher accuracy and stability in external verification. Thus, we confirm that the model is better at predicting the incidence of ESRD than Scr at baseline, suggesting that renal histology is helpful for predicting renal prognosis.

As a multicenter retrospective study, there are some limitations in our work. Despite no significant difference in clinical manifestations between patients with and without pathology, patients without renal pathological examination may cause bias in the prediction model. Due to the rarity of pediatric AAV, its sample size may not be large enough to effectively evaluate the predictive model, while the risk factors are limited to clinical and pathological indicators. Our team intends to design prospective, larger-scale studies and novel prognostic biomarkers to modify or develop more suitable models. Based on the available data, we established a predictive model of renal outcomes in AAGN rather than a model of risk of kidney injury in AAV. Considering that early diagnosis and early intervention are crucial for improving pediatric AVV, we should design prospective research to develop suitable models to predict renal injury in AAV using multiomics methods, artificial intelligence algorithms, and novel prognostic biomarkers, such as soluble CD206 [25].

In summary, this study describes the characteristics of the largest pediatric AAV cohort thus far, in which the incidence of renal involvement is over 90%, and more than half of patients developed ESRD. We also propose a predictive model of ESRD in AAV children based on the percentage of global glomerulosclerosis and eGFR at renal biopsy to fulfill the precise therapeutic strategy.

### Supplementary Information

Below is the link to the electronic supplementary material.

Supplementary file 1 (PDF 177 kb)

## Data Availability

The datasets generated during and analyzed during the current study are not publicly available due to privacy and ethical restrictions, but are available from the corresponding author on reasonable request.

## References

[1] Calatroni M, Consonni F, Allinovi M, Bettiol A, Jawa N, Fiasella S (2021). Prognostic factors and long-term outcome with ANCA-associated kidney vasculitis in childhood. Clin J Am Soc Nephrol.

[2] Watts RA, Mahr A, Mohammad AJ, Gatenby P, Basu N, Flores-Suárez LF (2015). Classification, epidemiology and clinical subgrouping of antineutrophil cytoplasmic antibody (ANCA)-associated vasculitis. Nephrol Dial Transplant.

[3] Jariwala M, Laxer RM (2020). Childhood GPA, EGPA, and MPA. Clin Immunol.

[4] de Joode AA, Sanders JS, Stegeman CA (2013). Renal survival in proteinase 3 and myeloperoxidase ANCA-associated systemic vasculitis. Clin J Am Soc Nephrol.

[5] Khalighi MA, Wang S, Henriksen KJ, Bock M, Keswani M, Chang A (2015). Pauci-immune glomerulonephritis in children: a clinicopathologic study of 21 patients. Pediatr Nephrol.

[6] Kouri AM, Andreoli SP (2017). Clinical presentation and outcome of pediatric ANCA-associated glomerulonephritis. Pediatr Nephrol.

[7] Berden AE, Ferrario F, Hagen EC, Jayne DR, Jennette JC, Joh K (2010). Histopathologic classification of ANCA-associated glomerulonephritis. J Am Soc Nephrol.

[8] Hirano D, Ishikawa T, Inaba A, Sato M, Shinozaki T, Iijima K (2019). Epidemiology and clinical features of childhood-onset anti-neutrophil cytoplasmic antibody-associated vasculitis: a clinicopathological analysis. Pediatr Nephrol.

[9] Watts R, Lane S, Hanslik T, Hauser T, Hellmich B, Koldingsnes W (2007). Development and validation of a consensus methodology for the classification of the ANCA-associated vasculitides and polyarteritis nodosa for epidemiological studies. Ann Rheum Dis.

[10] Jennette JC (2013). Overview of the 2012 revised International Chapel Hill Consensus Conference nomenclature of vasculitides. Clin Exp Nephrol.

[11] Pierrat A, Gravier E, Saunders C, Caira MV, Aït-Djafer Z, Legras B (2003). Predicting GFR in children and adults: a comparison of the Cockcroft-Gault, Schwartz, and modification of diet in renal disease formulas. Kidney Int.

[12] Dolezalova P, Price-Kuehne FE, Özen S, Benseler SM, Cabral DA, Anton J (2013). Disease activity assessment in childhood vasculitis: development and preliminary validation of the Paediatric Vasculitis Activity Score (PVAS). Ann Rheum Dis.

[13] Sinico RA, Di Toma L, Radice A (2013). Renal involvement in anti-neutrophil cytoplasmic autoantibody associated vasculitis. Autoimmun Rev.

[14] Rovin BH, Adler SG, Barratt J, Bridoux F, Burdge KA, Chan TM (2021). Executive summary of the KDIGO 2021 guideline for the management of glomerular diseases. Kidney Int.

[15] Sacri AS, Chambaraud T, Ranchin B, Florkin B, Sée H, Decramer S (2015). Clinical characteristics and outcomes of childhood-onset ANCA-associated vasculitis: a French nationwide study. Nephrol Dial Transplant.

[16] Morishita KA, Moorthy LN, Lubieniecka JM, Twilt M, Yeung RSM, Toth MB (2017). Early outcomes in children with antineutrophil cytoplasmic antibody-associated vasculitis. Arthritis Rheumatol.

[17] Cabral DA, Canter DL, Muscal E, Nanda K, Wahezi DM, Spalding SJ (2016). Comparing presenting clinical features in 48 children with microscopic polyangiitis to 183 children who have granulomatosis with polyangiitis (Wegener's): an ARChiVe cohort study. Arthritis Rheumatol.

[18] Yang J, Yang Y, Xu Y, Zhou L, Zhou L, Yin X (2022). Clinical and renal histology findings and different responses to induction treatment affecting the long-term renal outcomes of children with ANCA-associated vasculitis: a single-center cohort analysis. Front Immunol.

[19] Wu J, Pei Y, Rong L, Zhuang H, Zeng S, Chen L (2021). Clinicopathological analysis of 34 cases of primary antineutrophil cytoplasmic antibody-associated vasculitis in Chinese children. Front Pediatr.

[20] Day CJ, Howie AJ, Nightingale P, Shabir S, Adu D, Savage CO (2010). Prediction of ESRD in pauci-immune necrotizing glomerulonephritis: quantitative histomorphometric assessment and serum creatinine. Am J Kidney Dis.

[21] Huang S, Shen Q, Yang R, Lai H, Zhang J (2018). An evaluation of the 2010 histopathological classification of anti-neutrophil cytoplasmic antibody (ANCA)-associated glomerulonephritis: a Bayesian network meta-analysis. Int Urol Nephrol.

[22] Li ZY, Gou SJ, Chen M, Zhao MH (2013). Predictors for outcomes in patients with severe ANCA-associated glomerulonephritis who were dialysis-dependent at presentation: a study of 89 cases in a single Chinese center. Semin Arthritis Rheum.

[23] Hommos MS, Zeng C, Liu Z, Troost JP, Rosenberg AZ, Palmer M (2018). Global glomerulosclerosis with nephrotic syndrome; the clinical importance of age adjustment. Kidney Int.

[24] Brix SR, Noriega M, Tennstedt P, Vettorazzi E, Busch M, Nitschke M (2018). Development and validation of a renal risk score in ANCA-associated glomerulonephritis. Kidney Int.

[25] Aendekerk JP, Jiemy WF, Raveling-Eelsing E, Bijnens N, Abdul-Hamid MA, Strating IM (2022). CD163 and CD206 expression define distinct macrophage subsets involved in active ANCA-associated glomerulonephritis. J Autoimmun.

