# Supplementary material for: Risk factors for renal outcomes in children with antineutrophil cytoplasmic antibody-associated vasculitis: a nationwide retrospective study in China
Source: World J Pediatr. 2023 Oct 19;20(5):506–16. doi: 10.1007/s12519-023-00753-3 (PMC11136751; doi:10.1007/s12519-023-00753-3)
Supplement: Supplementary file 2 — Supplementary file 1 (PDF 177 kb) [file 12519_2023_753_MOESM1_ESM.pdf]

Supplementary Table 1. Enrollment of each center

Centers	City	Number of cases
Department of Nephrology, Children's Hospital of Fudan University	Shanghai	25
Department of Pediatrics, Jinling Hospital, Nanjing Medical University	Nanjing	24
Department of Pediatric Nephrology and Rheumatology, the First Affiliated Hospital of Sun Yat-sen University	Guangzhou	22
Department of Nephrology, Children’s Hospital of Chongqing Medical University	Chongqing	21
Department of Pediatrics, the Second Xiangya Hospital of Central South University	Changsha	16
Department of Pediatrics, Peking University First Hospital	Beijing	12
Department of Nephrology, Children's Hospital of Nanjing Medical University	Nanjing	11
Department of Nephrology, Beijing Children's Hospital	Beijing	10
Department of Pediatrics, Shengjing Hospital of China Medical University	Shenyang	7
Department of Nephrology and Immunology, Children’s Hospital of Soochow University	Suzhou	7
Department of Nephrology, the Children Hospital of Zhejiang University School of Medicine	Hangzhou	6
Department of Nephrology, Wuhan Children's Hospital	Wuhan	6
Department of Nephrology and Rheumatology, Shanghai Children's Hospital	Shanghai	5
Department of Nephrology and Immunology, Guiyang Maternal & Child Health Care Hospital	Guiyang	4
Department of Pediatrics, the First Affiliated Hospital Pediatrics of Zhengzhou University	Zhengzhou	3
Department of Pediatric Nephrology, Chengdu Women's and Children's Central Hospital	Chengdu	2
Department of Rheumatology and Immunology, Shenzhen Children's Hospital	Shenzhen	2

Supplementary Table 2. Demographic characteristics of 179 children with AAV at baseline

**Items**	**All (*N* = 179)**	**Follow-up patients (*n* = 114)**	**ESRD (*n* = 68)**	**Non-ESRD (*n* = 46)**	***P***
Age at diagnosis (y), median (IQR)	10.10 (8.00-12.08)	10.8 (8.25-12.25)	11.00 (9.75-13.00)	9.59 (7.00-12.00)	0.017
Time between onset and diagnosis (mon), median (IQR)	1.00 (0.05-3.40)	0.90 (0.47-2.27)	0.90 (0.40-2.47)	0.90 (0.48-2.17)	0.995
Male, *n* (%)	32 (17.9)	20 (17.5)	13 (19.1)	7 (15.2)	0.289
MPA, *n* (%)	136 (76.0)	87 (76.3)	54 (79.4)	33 (71.7)	Reference
GPA, *n* (%)	14 (7.8)	10 (8.8)	6 (8.8)	4 (8.7)	1.000
Unclassifiable vasculitis, *n* (%)	29 (16.2)	17 (14.9)	8 (11.7)	9 (19.6)	0.287

AAV antineutrophil cytoplasmic antibody-associated vasculitis, *ESRD* end-stage renal disease, *MPA* microscopic polyangiitis, *GPA* granulomatosis with polyangiitis, *IQR* interquartile range

Supplementary Table 3. Clinical characteristics of 179 children with and without follow-up at baseline

**Parameters**	**All (*N* = 179)**	**Follow-up patients (*n* = 114)**	**Loss of follow-up patients (*n* = 65)**	***P***
Age at diagnosis (y), median (IQR)	10.10 (8.00-12.08)	10.87 (8.25-12.25)	10.00 (7.25-12.29)	0.498
Male, *n* (%)	32 (17.9)	20 (17.5)	12 (18.5)	0.878
MPA, *n* (%)	136 (76.0)	87 (76.3)	49 (75.3)	Reference
GPA, *n* (%)	14 (7.8)	10 (8.8)	4 (6.2)	0.771
Unclassifiable vasculitis, *n* (%)	29 (16.2)	17 (14.9)	12 (18.5)	0.425
ANCA by ELISA, *n* (%)				
MPO-ANCA	139 (77.6)	91 (79.8)	48 (73.8)	Reference
PR3-ANCA	15 (8.4)	8 (7.0)	7 (10.8)	0.254
PR3-ANCA and MPO-ANCA	5 (2.8)	4 (3.5)	1 (1.5)	0.662
Negative	9 (5.1)	5 (4.4)	4 (6.2)	0.720
Missing	11 (6.1)	6 (5.3)	5 (7.7)	0.520
ANCA by IF, *n* (%)				
p-ANCA	108 (60.3)	71 (62.3)	37 (56.9)	Reference
c-ANCA	8 (4.5)	5 (4.4)	3 (4.6)	1.000
c-ANCA and p-ANCA	4 (2.2)	2 (1.8)	2 (3.1)	0.609
Negative	19 (10.6)	11 (9.6)	8 (12.3)	0.510
Missing	40 (22.3)	25 (21.9)	15 (23.1)	0.714
24-h urinary protein (g/d), median (IQR)	1.31 (0.64-2.29)	1.49 (0.86-2.75)	0.88 (0.32-1.98)	0.002
Scr (μmol/L), median (IQR)	195.00 (46.00-628.30)	426.40 (79.55-721.00)	51.45 (35.23-261.15)	< 0.001
eGFR (mL/min/1.73 m^2), median (IQR)	33.30 (11.20-135.60)	15.60 (10.45-77.20)	103.35 (30.10-175.90)	< 0.001

PVAS, median (IQR)	13.00 (12.00-18.00)	14.00 (12.00-18.00)	12.00 (10.50-16.50)	0.088
Renal involvement, *n* (%)	171 (95.5)	111 (97.4)	60 (92.3)	0.115
Edema, *n* (%)	84 (46.9)	59 (51.8)	25 (38.5)	0.087
Proteinuria, *n* (%)	160 (89.4)	108 (94.7)	52 (80.0)	0.002
Nephrotic proteinuria, *n* (%)	53 (29.6)	39 (34.2)	14 (21.5)	0.074
Hematuria, *n* (%)	161 (89.9)	105 (92.1)	57 (87.7)	0.579
RPGN, *n* (%)	58 (32.4)	39 (34.2)	19 (29.2)	0.494
Cutaneous involvement, *n* (%)	41 (22.9)	26 (22.8)	15 (23.1)	0.967
Respiratory system, *n* (%)	32 (17.9)	18 (15.8)	14 (21.5)	0.334
ENT, *n* (%)	14 (7.8)	10 (8.8)	4 (6.2)	0.530

MPA microscopic polyangiitis, *GPA* granulomatosis with polyangiitis, *ANCA* antineutrophil cytoplasmic antibody, *ELISA* enzyme-linked immunosorbent assay, *IF* immunofluorescence, *MPO-ANCA* myeloperoxidase-ANCA, *PR3-ANCA* proteinase 3-ANCA, *Scr* serum creatinine, *eGFR* estimated glomerular filtration rate, *PVAS* pediatric vasculitis activity score, *RPGN* rapidly progressive glomerulonephritis, *ENT* ear-nose-throat, *IQR* interquartile range

Supplementary Table 4. Clinical manifestations of 179 children with AAV at baseline

Systems	All (N = 179)	Follow-up patients (n = 114)	ESRD (n = 68)	Non-ESRD (n = 46)	P
Renal involvement	171 (95.5)	111 (97.4)	68 (100.0)	43 (93.5)	0.063
Edema	84 (46.9)	59 (51.8)	45 (66.2)	14 (30.4)	< 0.001
Proteinuria	160 (89.4)	108 (94.7)	66 (97.1)	42 (91.3)	0.219
Nephrotic proteinuria	53 (29.6)	39 (34.2)	26 (38.2)	13 (28.3)	0.271
Hematuria	161 (89.9)	105 (92.1)	64 (94.1)	41 (89.1)	0.481
Gross hematuria	51 (28.5)	37 (32.5)	20 (29.4)	17 (37.0)	0.399
Microscopic hematuria	110 (61.5)	68 (59.6)	44 (64.7)	24 (52.2)	0.243
RPGN	58 (32.4)	39 (34.2)	29 (42.6)	10 (21.7)	0.021
Constitutional symptom	68 (37.9)	36 (31.6)	20 (29.4)	16 (34.8)	0.545
Fever	43 (24.0)	25 (21.9)	14 (20.6)	11 (23.9)	0.818
Musculoskeletal involvement	31 (17.9)	13 (11.4)	7 (10.3)	6 (13.0)	0.766
Weight loss	7 (3.9)	2 (1.8)	1 (1.5)	1 (2.2)	0.404
Cutaneous involvement	41 (22.9)	26 (22.8)	13 (19.1)	13 (28.3)	0.254
Erythra	20 (11.1)	13 (11.4)	5 (7.4)	8 (17.4)	0.134
Purpura	20 (11.1)	11 (9.6)	7 (10.3)	4 (8.7)	1.000
Other skin involvement	10 (5.6)	5 (4.4)	2 (2.9)	3 (6.5)	0.391
Respiratory system	32 (17.9)	18 (15.8)	11 (16.2)	7 (15.2)	0.890
Massive hemoptysis/alveolar hemorrhage	26 (14.5)	16 (14.0)	10 (14.7)	6 (13.0)	1.000
Dyspnea	12 (6.7)	7 (6.1)	2 (2.9)	5 (10.9)	0.116
Respiratory failure	7 (3.9)	3 (2.6)	2 (2.9)	1 (2.2)	1.000
Abdomen	29 (16.2)	18 (15.8)	10 (14.7)	8 (17.4)	0.700
Abdominal pain	27 (15.1)	18 (15.8)	10 (14.7)	8 (17.4)	0.795
Gastrointestinal bleeding	4 (2.2)	2 (1.8)	0 (0)	2 (4.3)	0.161
Nervous system	19 (10.6)	15 (13.2)	12 (17.6)	3 (6.5)	0.085
ENT	14 (7.8)	10 (8.8)	6 (8.8)	4 (8.7)	1.000
Mucous membranes/eyes	10 (5.6)	2 (1.8)	2 (2.9)	0 (0)	0.514
Cardiovascular	4 (2.2)	2 (1.8)	1 (1.5)	1 (2.2)	1.000

Data are presented as n (%). *AAV* antineutrophil cytoplasmic antibody-associated vasculitis, *ESRD* end-stage renal disease, *RPGN* rapidly progressive glomerulonephritis, *ENT* ear-nose-throat

Supplementary Table 5. Clinical characteristics of 179 children with and without renal pathology at baseline

Parameters	All (*N* = 179)	Patients with renal pathology (*n* = 106)	Patients without renal pathology (*n* = 73)	*P*
Age at diagnosis (y), median (IQR)	10.10 (8.00-12.08)	10.86 (8.00-13.00)	10.00 (7.00-12.04)	0.271
Male, *n* (%)	32 (17.9)	16 (15.1)	16 (21.9)	0.242
MPA, *n* (%)	136 (76.0)	79 (74.5)	57 (78.1)	Reference
GPA, *n* (%)	14 (7.8)	9 (8.5)	5 (6.8)	0.654
Unclassifiable vasculitis, *n* (%)	29 (16.2)	18 (17.0)	11 (15.1)	0.663
ANCA by ELISA, *n* (%)				
MPO-ANCA	139 (77.6)	82 (77.3)	57 (78.0)	Reference
PR3-ANCA	15 (8.4)	8 (7.5)	7 (9.6)	0.673
PR3-ANCA and MPO-ANCA	5 (2.8)	4 (3.8)	1 (1.4)	0.648
Negative	9 (5.1)	4 (3.8)	5 (6.8)	1.000
Missing	11 (6.1)	4 (3.8)	7 (9.6)	1.000
ANCA by IF, *n* (%)				
p-ANCA	108 (60.3)	61 (57.5)	47 (64.4)	Reference
c-ANCA	8 (4.5)	7 (6.6)	1 (1.4)	0.137
c-ANCA and p-ANCA	4 (2.2)	2 (1.9)	2 (2.7)	1.000
Negative	19 (10.6)	12 (11.3)	7 (9.6)	0.587
Missing	40 (22.3)	24 (22.6)	16 (21.9)	0.701
24-h urinary protein(g/d), median (IQR)	1.31 (0.64-2.29)	1.40 (0.73-2.62)	1.21 (0.39-1.99)	0.214
Scr (μmol/L), median (IQR)	195.00 (46.00-628.30)	195.00 (51.45-577.00)	191.50 (42.00-685.75)	0.999
eGFR (mL/min/1.73	33.30 (11.20-	35.50 (11.45-114.85)	25.30 (11.10-	0.986

m²), median (IQR)	135.60)		153.10)	
PVAS, median (IQR)	13.00 (12.00-18.00)	14.00 (12.00-18.00)	13.00 (12.00-18.00)	0.569
Renal involvement, *n* (%)	171 (95.5)	104 (98.1)	67 (91.8)	0.064
Edema, *n* (%)	84 (46.9)	50 (47.2)	34 (46.6)	0.938
Proteinuria, *n* (%)	160 (89.4)	100 (94.3)	60 (82.2)	0.010
Nephrotic proteinuria, *n* (%)	53 (29.6)	37 (34.9)	16 (21.9)	0.061
Hematuria, *n* (%)	161 (89.9)	98 (92.5)	63 (86.3)	0.179
RPGN, *n* (%)	58 (32.4)	39 (34.2)	19 (26.0)	0.130
Cutaneous involvement, *n* (%)	41 (22.9)	22 (20.8)	19 (26.0)	0.409
Respiratory system, *n* (%)	32 (17.9)	20 (18.9)	12 (16.4)	0.667
ENT, *n* (%)	14 (7.8)	8 (7.5)	6 (8.2)	0.869

MPA microscopic polyangiitis, *GPA* granulomatosis with polyangiitis, *ANCA* antineutrophil cytoplasmic antibody, *ELISA* enzyme-linked immunosorbent assay, *MPO-ANCA* myeloperoxidase-ANCA, *PR3-ANCA* proteinase 3-ANCA, *IF* immunofluorescence, *Scr* serum creatinine, *eGFR* estimated glomerular filtration rate, *PVAS* pediatric vasculitis activity score, *RPGN* rapidly progressive glomerulonephritis, *ENT* ear-nose-throat, *IQR* interquartile range

Supplementary Table 6. Renal pathological features, clinical manifestations and laboratory parameters of renal biopsy

**Items**	**All (N = 106)**
Time from onset to diagnosis (mon), median (IQR)	0.79 (0.33-2.24)
Edema, *n* (%)	50 (47.2)
Proteinuria, *n* (%)	100 (94.3)
Hematuria, *n* (%)	98 (92.5)
Gross hematuria	31 (29.2)
Microscopic hematuria	67 (63.2)
Oliguria/anuria, *n* (%)	26 (25.5)
Hemodialysis, *n* (%)	41 (38.7)
WBC ($\times 10^9$/L), median (IQR)	8.60 (6.44-11.65)
Hemoglobin (g/L), mean ± SD	86.24 ± 22.30
Platelet ($\times 10^9$/L), median (IQR)	280.00 (195.50-357.50)
CRP (mg/L), median (IQR)	6.00 (2.86-25.00)
BUN (mmol/L), median (IQR)	14.95 (6.11-27.11)
Scr (μmol/L), median (IQR)	175.60 (57.00-452.00)
eGFR (mL/min/1.73 m^2), median (IQR)	36.93 (15.24-112.32)
Albumin (g/L), median (IQR)	34.25 (29.40-38.10)
IgG (g/L), median (IQR)	8.94 (6.52-11.70)
C3 (g/L), median (IQR)	0.95 (0.69-1.13)
24-h urinary protein (g/d), median (IQR)	1.40 (0.71-2.10)
Normal glomerulus (%), median (IQR)	18.35 (0.00-46.98)
Crescent (%), median (IQR)	35.35 (12.28-68.25)
Cellular crescent (%)	8.25 (0.00-25.00)
Fibrocellular crescent (%)	11.00 (0.00-38.00)
Fibrous crescent (%)	0.00 (0.00-8.09)
Global glomerulosclerosis (%), median (IQR)	11.80 (0.00-41.33)
Segmental glomerulosclerosis (%), median (IQR)	0.00 (0.00-4.90)
Granuloma and necrosis, *n* (%)	3 (2.8)
Interstitial inflammatory cell infiltration, *n* (%)	93 (87.7)
Interstitial fibrosis, *n* (%)	56 (52.8)
Tubular atrophy, *n* (%)	64 (60.4)

WBC white blood cell, *CRP* C-reactive protein, *BUN* blood urea nitrogen, *Scr* serum creatine, *eGFR* estimated glomerular filtration rate, *IgG* immunoglobulin G, *IQR* interquartile range

Supplementary Table 7. Demographic characteristics of 32 children with eGFR > 60 mL/min/1.73 m^2 at baseline

**Items**	**Follow-up patients (*n* = 32)**	**ESRD (*n* = 5)**	**Non-ESRD (*n* = 27)**	***P***
Age at diagnosis (y), median (IQR)	8.96 (6.00-10.32)	4.00 (2.00-11.71)	9.00 (7.00-10.00)	0.380
Time between onset and diagnosis (mon), median (IQR)	0.93 (0.67-3.80)	8.00 (2.87-25.00)	0.92 (0.65-2.08)	0.034
Male, *n* (%)	7 (21.9)	2 (40.0)	5 (18.5)	0.296
MPA, *n* (%)	19 (59.4)	2 (40.0)	17 (63.0)	Reference
GPA, *n* (%)	6 (18.8)	3 (60.0)	3 (11.1)	0.070
Unclassifiable vasculitis, *n* (%)	7 (21.9)	0 (0)	7 (25.9)	1.000

eGFR estimated glomerular filtration rate, *ESRD* end-stage renal disease, *MPA* microscopic polyangiitis, *GPA* granulomatosis with polyangiitis, *IQR* interquartile range

Supplementary Table 8. Clinical manifestations of 32 children with eGFR > 60 mL/min/1.73 m^2 at baseline

Systems	Follow-up patients (*n* = 32)	ESRD (*n* = 5)	Non-ESRD (*n* = 27)	*P*
Renal involvement	29 (90.6)	5 (100)	24 (88.9)	1.000
Edema	6 (18.8)	1 (20)	5 (18.5)	1.000
Proteinuria	28 (87.5)	5 (100)	23 (85.2)	1.000
Hematuria	28 (87.5)	5 (100)	23 (85.2)	1.000
RPGN	2 (6.3)	1 (20)	1 (3.7)	0.292
Constitutional symptom	12 (37.5)	2 (40)	10 (37.0)	1.000
Fever	9 (28.1)	2 (40)	7 (25.9)	0.604
Musculoskeletal involvement	4 (12.5)	1 (20)	3 (11.1)	0.512
Cutaneous involvement	13 (40.6)	3 (60)	10 (37.0)	0.374
Erythra	8 (25.0)	2 (40)	6 (22.2)	0.578
Purpura	4 (12.5)	1 (20)	3 (11.1)	0.512
Other skin involvement	2 (6.3)	0 (0)	2 (7.4)	0.619
Respiratory system	9 (28.1)	2 (40)	7 (25.9)	0.604
Abdomen	5 (15.6)	0 (0)	5 (18.5)	0.564
ENT	5 (15.6)	2 (40)	3 (11.1)	0.163
Nervous system	1 (3.1)	1 (20)	0 (0)	0.156
Mucous membranes/eyes	1 (3.1)	1 (20)	0 (0)	0.156
Cardiovascular	1 (3.1)	0 (0)	1 (3.7)	1.000

Data are presented as *n* (%). *eGFR* estimated glomerular filtration rate, *ESRD* end-stage renal disease, *RPGN* rapidly progressive glomerulonephritis, *ENT* ear-nose-throat

Supplementary Table 9. Laboratory parameters of 32 children with eGFR > 60 mL/min/1.73 m^2 at baseline

**Parameters**	**Follow-up patients (*n* = 32)**	**ESRD (*n* = 5)**	**Non-ESRD (*n* = 27)**	***P***
WBC (× 10^9/L), median (IQR)	8.34 (5.98-14.72)	9.45 (7.28-12.15)	8.34 (5.70-14.88)	0.842
Hemoglobin (g/L), median (IQR)	102.50 (72.25-122.75)	81.00 (48.50-139.00)	104.00 (79.00-122.00)	0.911
Platelet (× 10^9/L), median (IQR)	374.00 (291.25-466.50)	358.50 (272.25-450.75)	380.50 (291.25-467.00)	0.617
Albumin (g/L), median (IQR)	39.53 (36.65-42.12)	35.45 (24.23-42.25)	39.53 (36.70-42.12)	0.586
C3 (g/L), median (IQR)	1.10 (0.85-1.22)	1.36 (1.06-1.59)	1.09 (0.80-1.18)	0.080
ANCA by ELISA, *n* (%)				
MPO-ANCA	22 (68.8)	5 (100)	17 (63.0)	Reference
PR3-ANCA	2 (6.3)	0 (0)	2 (7.4)	1.000
PR3-ANCA and MPO-ANCA	2 (6.3)	0 (0)	2 (7.4)	1.000
Negative	3 (9.4)	0 (0)	3 (11.1)	1.000
Missing	3 (9.4)	0 (0)	3 (11.1)	1.000
ANCA by IF, *n* (%)				
p-ANCA	17 (53.1)	5 (100)	12 (44.4)	Reference
c-ANCA	1 (3.1)	0 (0)	1 (3.7)	1.000
c-ANCA and p-ANCA	0 (0)	0 (0)	0 (0)	-
Negative	3 (9.4)	0 (0)	3 (11.1)	0.539
Missing	11 (34.4)	0 (0)	11 (40.7)	0.125
SBP, median (IQR)	105.00 (98.00-119.00)	101.50 (95.00-117.75)	105.00 (99.00-119.00)	0.660
DBP, median (IQR)	69.00 (63.00-76.00)	67.00 (58.00-85.00)	69.00 (63.00-76.00)	0.989
24-h urinary protein (g/d), median (IQR)	1.00 (0.36-1.67)	0.71 (0.29-2.28)	1.13 (0.49-1.67)	0.712
Number of RBC in urine routines (/μL), median (IQR)	105.70 (23.50-456.85)	35.00 (19.00-1914.52)	111.00 (25.00-432.40)	1.000
BUN (mmol/L), median (IQR)	5.58 (3.10-7.08)	7.48 (6.06-10.93)	4.80 (2.89-6.95)	0.082
Scr (μmol/L), median (IQR)	45.00 (34.18-66.20)	48.00 (39.90-70.55)	42.00 (32,30-67.00)	0.579
eGFR (mL/min/1.73 m^2), median (IQR)	138.90 (92.40-182.00)	133.10 (127.60-138.60)	138.90 (90.40-184.95)	0.681
PVAS, median (IQR)	13.50 (10.00-16.75)	16.00 (11.50-22.50)	12.00 (10.00-17.00)	0.352

eGFR estimated glomerular filtration rate, *ESRD* end-stage renal disease, *WBC* white blood cell, *ANCA* anti-neutrophil cytoplasmic antibodies, *ELISA* enzyme-linked immunosorbent assay, *MPO-ANCA* myeloperoxidase-ANCA, *PR3-ANCA* proteinase 3-ANCA, *IF* immunofluorescence, *SBP* systolic blood pressure, *DBP* diastolic blood pressure, *RBC* red blood cell, *BUN* blood urea nitrogen, *Scr* serum creatine, *PVAS* pediatric vasculitis activity score, *IQR* interquartile range

Supplementary Table 10. Clinical characteristics of patients in validation of Scr at baseline

**Items**	**Follow-up patients (*n* = 134)**	**Internal (*n* = 114)**	**External (*n* = 20)**	***P***
Age at diagnosis (y), median (IQR)	10.83 (8.00-12.27)	10.87 (8.25-12.25)	10.50 (7.19-12.48)	0.603
Male, *n* (%)	26 (19.4)	20 (17.5)	6 (30.0)	0.222
MPA, *n* (%)	102 (76.7)	87 (76.3)	16 (80.0)	Reference
GPA, *n* (%)	12 (9.0)	10 (8.8)	2 (10.0)	1.000
Unclassifiable vasculitis, *n* (%)	19 (14.3)	17 (14.9)	2 (10.0)	0.736
ANCA by ELISA, *n* (%)				
MPO-ANCA	107 (80.5)	91 (79.8)	17 (85.0)	Reference
PR3-ANCA	11 (8.3)	8 (7.0)	3 (15.0)	0.392
PR3-ANCA and MPO-ANCA	4 (3.0)	4 (3.5)	0 (0)	1.000
Negative	5 (3.8)	5 (4.4)	0 (0)	1.000
Missing	6 (4.5)	6 (5.3)	0 (0)	0.589
BUN (mmol/L), median (IQR)	19.72 (8.67-31.50)	20.31 (8.58-31.66)	19.24 (10.53-27.80)	0.902
Scr (μmol/L), median (IQR)	357.10 (79.50-701.00)	426.40 (79.55-721.00)	216.95 (76.95-527.05)	0.352
eGFR at baseline (mL/min/1.73 m^2), median (IQR)	18.55 (10.88-78.40)	15.60 (10.45-77.20)	33.30 (13.86-104.31)	0.236
PVAS, median (IQR)	14.00 (12.00-18.00)	14.00 (12.00-18.00)	16.00 (12.00-21.00)	0.163
ESRD, *n* (%)	77 (57.5)	68 (59.6)	9 (45.0)	0.327

Scr serum creatine, *MPA* microscopic polyangiitis, *GPA* granulomatosis with polyangiitis, *ANCA* anti-neutrophil cytoplasmic antibodies, *ELISA* enzyme-linked immunosorbent assay, *MPO-ANCA* myeloperoxidase-ANCA, *PR3-ANCA* proteinase 3-ANCA, *BUN* blood urea nitrogen, *eGFR* estimated glomerular filtration rate, *PVAS* pediatric vasculitis activity score, *ESRD* end-stage renal disease, *IQR* interquartile range

Supplementary Table 11. Clinical characteristics of patients in validation of the predictive model based on eGFR and percentages of global glomerulosclerosis in renal biopsy

**Items**	**Follow-up patients (*n* = 81)**	**Internal validation (*n* = 61)**	**External validation (*n* = 20)**	***P***
Time from onset to renal biopsy (mon), median (IQR)	1.00 (0.40-2.00)	0.90 (0.40-2.14)	1.00 (0.33-1.93)	0.528
BUN (mmol/L), median (IQR)	16.50 (8.40-16.50)	16.60 (8.34-27.73)	15.90 (8.82-25.6)	0.961
Scr (μmol/L), median (IQR)	261.00 (83.25-499.15)	337.00 (90.55-545.83)	212.00 (81.00-377.00)	0.291
eGFR at renal biopsy (mL/min/1.73 m^2), median (IQR)	22.36 (15.01-79.15)	19.42 (12.90-75.24)	33.30 (18.63-102.08)	0.124
Global glomerulosclerosis (%), median (IQR)	9.00 (0.00-44.40)	11.80 (0.00-44.40)	0.00 (0.00-50.00)	0.261
Segmental glomerulosclerosis (%), median (IQR)	0.00 (0.00-3.80)	0.00 (0.00-5.40)	0.00 (0.00-17.64)	0.344

eGFR estimated glomerular filtration rate, *BUN* blood urea nitrogen, *Scr* serum creatine, *IQR* interquartile range
